# Embracing Sustainability: The World of Bio-Based Polymers in a Mini Review

**DOI:** 10.3390/polym16070950

**Published:** 2024-03-30

**Authors:** Grazia Isa C. Righetti, Filippo Faedi, Antonino Famulari

**Affiliations:** Department of Chemistry, Materials and Chemical Engineering “Giulio Natta”, Politecnico di Milano, Piazza Leonardo da Vinci 32, 20133 Milano, Italy

**Keywords:** bio-based polymers, polymers, sustainable materials, renewable sources, polymeric materials

## Abstract

The proliferation of polymer science and technology in recent decades has been remarkable, with synthetic polymers derived predominantly from petroleum-based sources dominating the market. However, concerns about their environmental impacts and the finite nature of fossil resources have sparked interest in sustainable alternatives. Bio-based polymers, derived from renewable sources such as plants and microbes, offer promise in addressing these challenges. This review provides an overview of bio-based polymers, discussing their production methods, properties, and potential applications. Specifically, it explores prominent examples including polylactic acid (PLA), polyhydroxyalkanoates (PHAs), and polyhydroxy polyamides (PHPAs). Despite their current limited market share, the growing awareness of environmental issues and advancements in technology are driving increased demand for bio-based polymers, positioning them as essential components in the transition towards a more sustainable future.

## 1. Introduction

The interest in polymer science and technology has witnessed an unprecedented growth over the past few decades, leading to rapid advancements in the development of polymers and plastics. Throughout the 20th century, the polymer industry was heavily reliant on petroleum-related chemistry, refineries, and processes. The petrochemical boom in the latter half of the century played a pivotal role in propelling the progress of the chemical sciences, experiencing unparalleled growth in the organic chemical industry.

Despite ongoing debates about the detrimental environmental impact of petroleum-related processes, industries hesitated to alter their raw materials or methodologies until the situation reached a critical juncture. Notably, this era saw the discovery of various monomers for polymerizations, propelling synthetic polymers to a prominent position in the global market, as they could be utilized in the production of a wide array of everyday commodities.

This remarkable scientific and technological upswing led to the gradual displacement of naturally occurring polymers by their synthetic counterparts. This choice was primarily driven by economic considerations, as the cost of oil was significantly lower than the processing of natural fibers at that time. Although polymers derived from renewable sources continued to exist, they played a minor role due to limited investments compared to the substantial funds allocated to petrochemistry.

However, the polymer industry copes with two significant challenges: global warming and the depletion of fossil resources. A potential solution to address these issues involves exploring the use of renewable sources instead of fossil-based ones. Moreover, the decreased availability of fossil resources resulted in a surge in market prices, exacerbated by the fact that oil is not universally accessible and requires certain states to be subjected to others. In contrast, renewable resources are globally shared, making them highly valuable [1,2].

In the ever-evolving landscape of polymers for materials science, the quest for sustainable alternatives has become imperative. In light of the growing concerns and an increased awareness of the environmental impact of traditional plastics, researchers are turning their attention to bio-based polymers as a promising solution. The urgency to reduce the consumption of fossil fuels, together with the negative impact of plastic pollution on the environment, has exponentially increased the interest in these alternative materials. As we try to face the challenges of a global environmental crisis, the significance of bio-based polymers extends beyond their material properties. Their potential to mitigate carbon emissions, reduce plastic pollution, and contribute to a circular economy underscores their pivotal role in building a sustainable future.

Bio-based polymers are derived from renewable resources such as plants, microbes, and agricultural or forestry feedstock and represent a paradigm shift in the pursuit of eco-friendly materials. They can be produced from the raw sources through chemical, physical, or biochemical transformations, and due to the sustainable nature of the starting material, bio-based polymers offer several advantages such as a reduced carbon footprint and the potential for higher biodegradability [3,4]. Even if it was logical to assume that polymers crafted from renewable and frequently biodegradable sources would possess biodegradable properties as well, this feature is not always assured. Variations in functional groups, crosslink density, and the inclusion of non-biodegradable co-monomers through copolymerization may lead to materials that do not necessarily demonstrate substantial biodegradability [5,6]. As the terms “biodegradable” and “bio-based” are often mentioned in the literature, it is crucial to underline the key difference between these two types of polymers. A biodegradable polymer is defined as a material able to undergo degradation and deterioration. The biodegradation of a polymer (bio-based or synthetic) is defined as the chemical decomposition process of the substance into environmentally friendly compounds [7]. After a first fragmentation step of the high molecular mass (HMW) polymer to a lower molecular mass (LMW) group of chains [8,9], the final degradation is dependent on microorganisms which, based on the environment (aerobic or anaerobic), will convert the substances mainly into CO_2_, H_2_O, and CH_4_ [10]. On the other hand, bio-based polymers are defined by IUPAC as “composed or derived in whole or in part of a biological product issued from biomass (including plant, animal, and marine or forestry materials)” [11].

The primary advantage of bio-based polymers relies on substituting non-renewable resources with renewable carbon sourced from biomass, a crucial step in achieving sustainability and promoting a climate-friendly plastics industry. Another key benefit is that biodegradable bio-based polymers constitute roughly a quarter of all bio-based polymers (depending on environmental conditions). These next-generation polymers might be the key to a lower reliance on fossil fuels, offering a potential solution to the problem of uncollected and unprocessed plastics in the environment [12,13].

**Figure 1:**
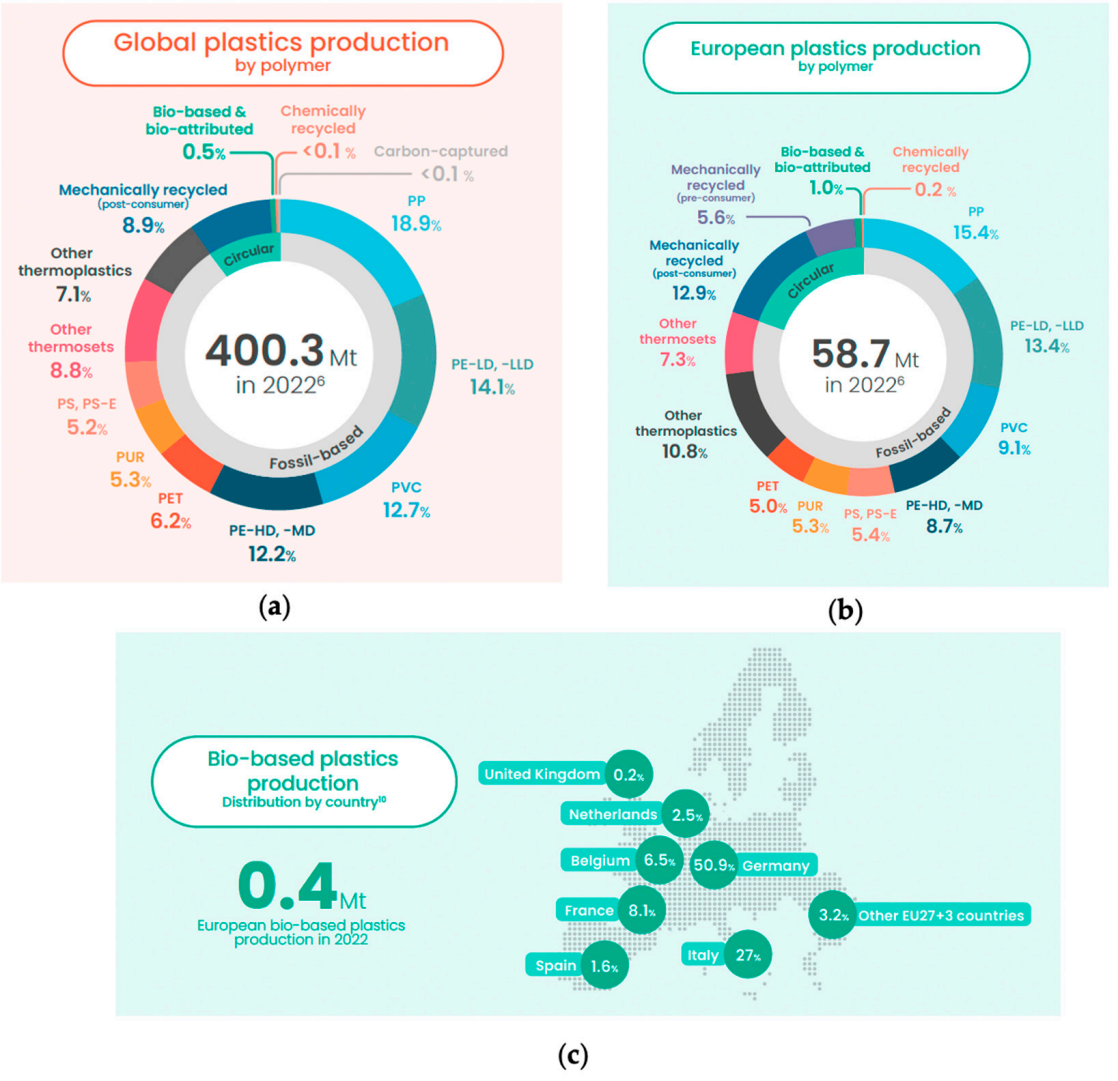
Plastic production in 2023: (**a**) global plastic production, (**b**) European plastic production, (**c**) bio-based plastic production per country in Europe. The credit for the graphs belongs to PlasticEurope and is attributed to the report *‘Plastic Europe—The Fast Facts 2023*’. The graphs have been reproduced with the permission of PlasticEurope [12].

Bio-based polymers still hold just a small fraction of the market, accounting for 0.5% of the overall global plastic production and 1.0% of the European market in 2023 (Figure 1) [14]. However, the global market request is estimated to expand at a compound annual growth rate of 18.8% from 2023 to 2030 [15].

**Figure 2:**
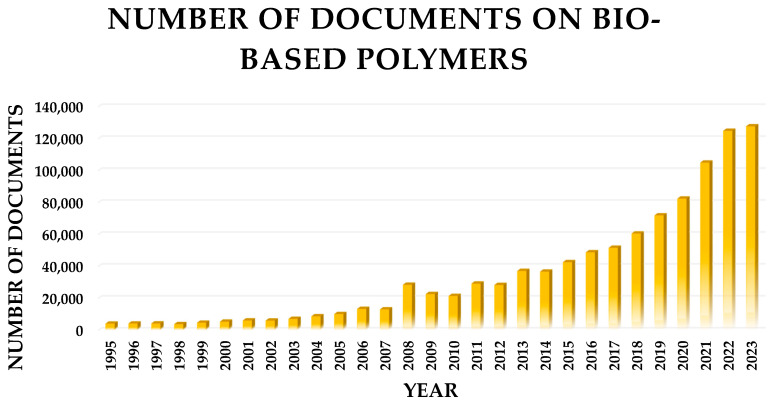
Total number of documents produced on bio-based polymers per year from 1995 to 2003, including both articles and patents. Dimension AI was used as a tool to retrieve the total number of documents per year on the given topic.

**Figure 3:**
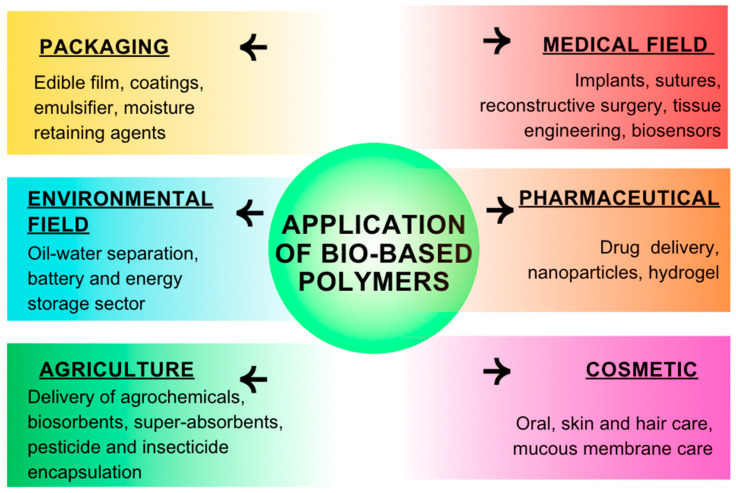
Examples of some applications of biopolymers and bio-based polymers.

Despite the currently low market fraction occupied by bio-based polymers, their crucial role in shifting plastic production towards a greener and more sustainable direction is underlined by the growing interest among researchers in both academia and industry. This is evident not only in the increasing number of scientific articles and citations dedicated to this subject (Figure 2) but also in the wide application of those polymers in different fields (Figure 3). 

This mini-review aims to provide an overview on three important bio-based polymers: (a) polylactic acid (PLA), (b) polyhydroxy alkanoates (PHA), and (c) polyhydroxy polyamides (PHPAs).

These three polymers were chosen due to their importance among all the biobased polymers. In fact, polylactic acid (PLA) and polyhydroxy alkanoates (PHA) are the most-studied bio-based polymeric materials due to their potential to replace petroleum-based polymeric materials thanks to their peculiar properties. Their importance is also underlined by the fact that they have already found applications in many fields, ranging from packaging to biomedical fields [16]. On the other hand, polyhydroxy polyamides (PHPAs) have also gained increasing attention with the aim of synthetizing new, more hydrophilic and degradable polymeric materials [17]. What makes this class of polymers highly attractive is the nature of the dicarboxylic acid employed, which is a natural carbohydrate-based monomeric building block that displays peculiar structural features (e.g., stereochemistry) that can help tune the polyamide’s final properties [18].

## 2. Bio-Based Polymers

### 2.1. Polylactic Acid

Polylactic acid (PLA), a biodegradable aliphatic polyester, has been known since 1845 when Theophile-Jules Pelouze first synthesized it [19]. It has been extensively studied in the past 20–25 years due to its exceptional properties, such as its high biodegradability and biocompatibility.

PLA is composed of lactic acid as its fundamental building block. This monomer is a hydroxyl carboxylic acid that can be obtained from renewable sources like corn starch (via fermentation processes) or sugarcane. Even if various renewable resources can be employed, corn stands out, as it offers the advantage of yielding a high-quality feedstock for fermentation, resulting in the production of high-purity lactic acid. Depending on the microbial strain utilized in the fermentation process, both l-lactic acid and d-lactic can be obtained [20].

#### 2.1.1. Polylactic Acid Synthesis

**Scheme 1:**
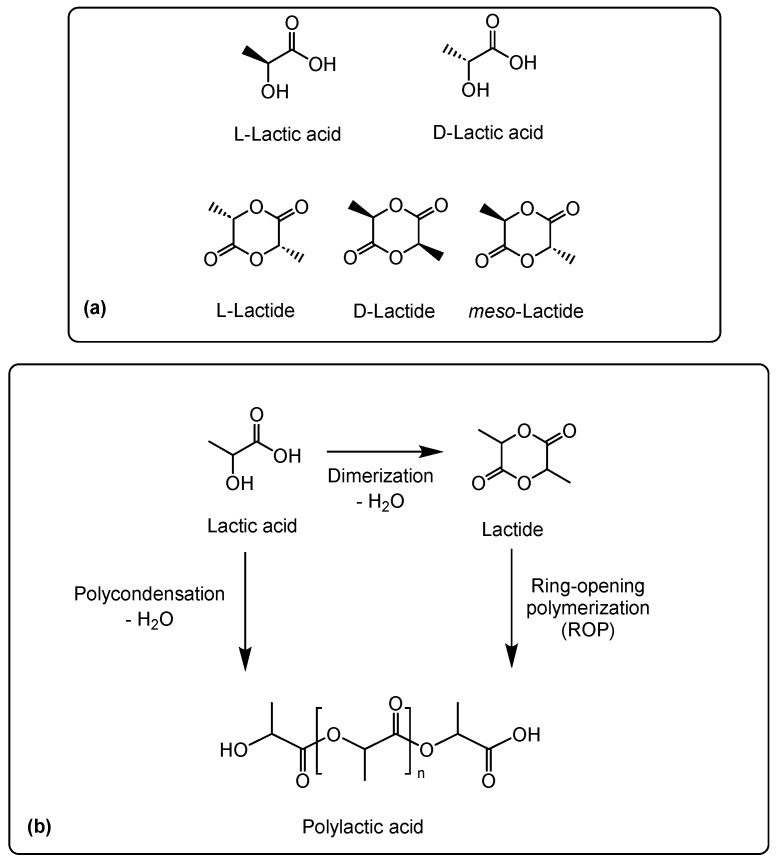
(**a**) optical monomers of lactic acid and stereoisomers of lactide; (**b**) formation of polylactic acid via polycondensation and ring-opening polymerization.

PLA can be synthetized via direct polymerization of lactic acid or via ring-opening polymerization (ROP) of the lactide monomer, where the lactide is the cyclic dimer of lactic acid (Scheme 1). Typically, the polycondensation process is associated with a low-molecular-weight polymeric product due to water formation associated with the lactic acid condensation process causing the degradation of the forming polymeric chain itself. Water removal systems and protocols utilizing high temperatures in the presence of catalysts have been proposed to attain higher degrees of polymerization in the production of polylactic acid (PLA) [21]. Nevertheless, these approaches still face limitations, primarily stemming from challenges in effectively removing water as the degree of polymerization (DP) of PLA increases, and from material degradation issues at elevated temperatures [22].

The current preferred synthetic pathway for producing polylactic acid (PLA) is ring-opening polymerization. This method offers enhanced control over various reaction parameters such as polydispersity, molecular weight, and stereochemistry. Notably, ring-opening polymerization allows for precise control over the insertion of lactide monomers, and the stereochemistry of PLA can be easily manipulated by polymerizing d, l, or meso-lactide monomers [23]. The ability to control and modulate stereochemistry is pivotal, as it enables the production of polymers with markedly different characteristics. From this perspective, the investigation of metal complexes as catalysts in lactide polymerization via coordination–insertion mechanisms has experienced a surge in interest over the past decade. While tin (II) bis(2-ethylhexanoate), commonly referred to as Sn(Oct)_2_, remains the most-utilized catalyst for industrial PLA synthesis, other examples such as aluminum, zinc, magnesium, and calcium have also gained widespread usage [24,25,26,27].

#### 2.1.2. Polylactic Acid: Physical Properties

Extensive research has focused on understanding the properties of polylactic acid (PLA). These properties are influenced by factors such as stereochemistry, processing temperature, annealing time, and molecular weight (Mw). Specifically, the stereochemistry and thermal history of PLA have a direct impact on its crystallinity, which in turn affects its properties.

Crystallinity, which refers to the proportion of crystalline regions in the polymer compared to the amorphous content, plays a crucial role in determining various properties of PLA. These properties include hardness, modulus, tensile strength, stiffness, crease resistance, and melting points (the values of some of these parameters are reported in Table 1, with those of polypropylene and polyethylene provided for a comparison with synthetic non-bio-based polymers). The degree of crystallinity significantly influences the mechanical, thermal, and processing characteristics of PLA, making it a key parameter in understanding and optimizing PLA-based materials for diverse applications.

PLA is renowned for its exceptional optical properties and high tensile strength. However, its versatility and potential applications might be compromised by its rigidity and brittleness at room temperature; characteristics that stem from its glass transition temperature (T_g_) of approximately 55 °C. PLA might meet the mechanical property requirements for most rigid objects, but it needs to be plasticized when it comes to its applications, especially as films in the packaging industry. Plasticizers play a vital role in enhancing the processability, flexibility, and ductility of polymers. In the case of semi-crystalline polymers like PLA, an effective plasticizer needs to not only reduce the glass transition temperature (T_g_) but also depress the melting temperature (T_m_) and overall crystallinity [30,39,40]. Different kinds of molecules are reported in the literature as plasticizing agents for PLA. Low-molecular-weight agents such as glycerol, sorbitol, bishydroxymethyl malonate (DBM), adipates and citrates [41], vegetable oils derivatives [42,43], and 10,16-dihydroxy hexadecenoic acid and its derivatives [29] have been tested, but these molecules showed a high mobility inside the PLA matrix due to their low molecular weight [44]. In order to reduce the plasticizer migration phenomena, blending PLA with higher-molecular-weight compounds such as polyethylene glycol (PEG) [45,46], poly(propylene) glycol (PPG) [47], or poly(diethylene adipate) (PDEA) [48] has emerged as an appealing solution. Some starch-blended PLA mixtures like Mater-Bi (Novamont, Novara, Italy) and Bioplast (Biotec, Emmerich am Rhein, Germany) [49] have also reached the market. Despite the wide range of plasticizer choices offered by the ever-growing scientific literature, this is often constrained by legislative or technical requirements that are specific to their application, especially in the medical and food industries [44,46,50]. This limitation makes the selection process more challenging. In fact, the plasticizer used for PLA must meet stringent criteria such as biodegradability, non-toxicity, and/or biocompatibility. As a result, the most common plasticizers used for PLA are low-molecular-weight polyethylene glycols (PEGs) [30,40,45,46].

#### 2.1.3. Polylactic Acid Applications

Currently, PLA-based products have entered the market with various applications, ranging from drug carriers, temporary implants, and bone-fixing elements to degradable dishes and packaging. PLA, as a highly versatile and environmentally friendly polymer, possesses renewability, biodegradability, transparency, colorlessness, processability, and mechanical properties, making it increasingly interesting as a substitute polymer for those products where recycling, reuse, and product recovery of the materials are not feasible [51]. As the production costs for PLA manufacturing processes decrease, the potential for PLA to be utilized in a broad array of products continues to grow.

One of the largest potential application areas for PLA is in fibers, where the first commercial success as a fiber material was in the form of resorbable sutures [23]. Since then, PLA has been extensively studied, especially for its potential use in other medical applications due to its bioresorbability and biocompatible properties in the human body. In this sector, PLA is employed not only in wound management but also to produce resorbable stents [52], as encapsulation systems to enhance target-specific drug delivery [53,54,55,56,57,58], in the orthopedic field as fracture fixation systems and as temporary fillings in facial reconstructive surgery [59], and in tissue engineering and regenerative medicine fields [30,60,61].

The second-largest application for PLA is in the production of films, particularly in the food packaging industry. PLA films are prized for their transparency and excellent deadfold or twist retention properties, making them increasingly sought after as a green alternative in food packaging due to their biodegradability. In the packaging sector, PLA finds applications ranging from containers for fresh produce to disposable cutlery and drinking cups. While PLA presents several advantages, such as biodegradability, it also poses challenges that need to be addressed. These include inferior moisture barrier properties and brittleness [62]. To overcome these challenges, various approaches are being explored, including coatings, lamination, blends with other polymers, and chemical or physical modifications [63]. However, achieving a balance between stiffness and toughness while maintaining a high biobased content remains a significant challenge. Strategies such as plasticizing PLA with its own monomers or blending it with other polymers have been attempted, but they often encounter issues such as phase separation [24,64].

PLA is a good candidate material for 3D printing technology, and in the last three years, many investigations have been focused on tuning its properties for this application [65]. Melt extrusion (MEX) is the most-used 3D molding method, and it imposes some requirements to the molding material such as a low viscosity, sufficient adhesion, robustness, and stability. PLA as a 3D printing material partially meets these requirements, but it presents some defects such as a poor high-temperature resistance, brittleness, and low impact resistance. The performance of PLA’s extruded filament can be improved by adding auxiliary material and changing the processing parameters. The research in this field has focused on changing the mechanical properties of PLA filaments and improving their printing performance. One promising root to make PLA suitable for 3D printing is to add natural fillers to the polymers, such as calcined shell particles (CSh), straw meal, and poplar fibers. The resulting materials are completely biodegradable and result in a higher tensile strength of the polymers [66,67,68]. Copper and lignin are used as fillers for impact resistance enhancements. The addition of lignin microspheres resulted in an increase in impact strength of 100% [69], and Cu-modified PLA achieved remarkable results in impact resistance tests as well [70]. Regarding the change in processing parameters, Fontana et al. analyzed the effects of layer height (LH) and fill rate on the ultimate tensile strength (UTS) values of 3D-printed tensile specimens of Makerbot PLA rigid materials [71,72]. The data analysis revealed that the LH had a more prominent effect on the mechanical strength than the fill rate.

The inherent ignitability properties of neat PLA restrict its application [71,72]. A way to improve PLA’s fire safety is to add flame retardants to the neat polymers. Among them, the most successful have been intumescent flame retardants (IFR), P/N synergistic systems, metal oxides, hydroxides, and nanomaterials [73,74,75].

Intumescent flame retardants (IFR) proved to be very efficient, environmentally friendly, and also capable of increasing the mechanical properties of PLA [76]. Recently, a novel bio-based IFR with a nanosheet structure, PP-Fe, was fabricated using self-assembly technology. The new material presented an outstanding increase in fire safety and UV resistance. For fire safety, introduction of 20.0 wt% PP-Fe nanosheets to PLA resulted in significant reductions in the PHRRc (72.7%), THRc (41.6%), and TSP (64.7%) values in the CCT and achieved a UL-94 V-0 rating with an LOI of 31.2%. The UPF value of the PLA/20PP-Fe composite was 120%, categorized as excellent UV shielding [77].

Despite these challenges, PLA holds promise as a future material for eco-friendly food packaging, and ongoing research and innovation in this field are expected to lead to further improvements and applications.

### 2.2. Polyhydroxy Alcanoates

Poly(hydroxyalkanoates) (PHAs) represent a class of biopolymers prized for their biodegradability and their ability to be derived from natural resources. Falling under the umbrella of aliphatic polyesters, PHAs have gained significant attention from both the scientific and industrial sectors owing to their potential as biomaterials. In fact, due to the combination of their innate biodegradability, natural origin, functional versatility, and compatibility with biological systems and their mechanical and thermal properties, they are viewed as promising alternatives to petroleum-based plastics [78]. Notably, PHA stands out as an ideal candidate due to its ability to form small pores, facilitating efficient recycling and boasting a high volume-to-surface ratio [79].

**Figure 4:**
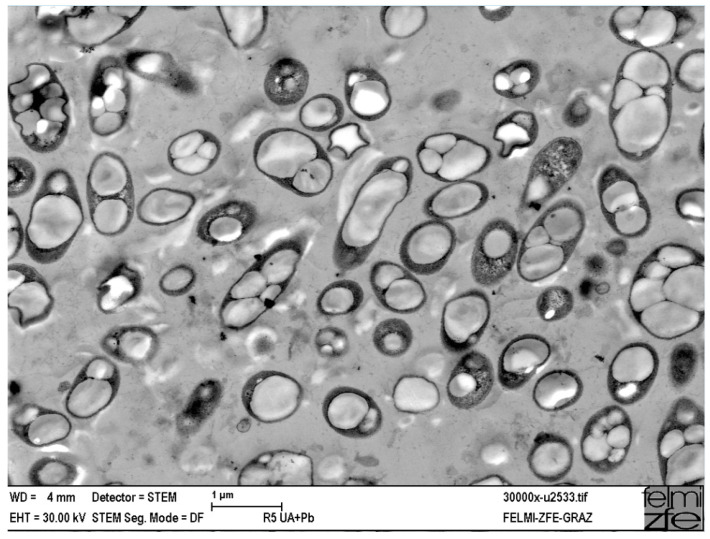
Cupriavidus necator DSM 545, a metalophilic strain, containing PHA granules (bright intracellular inclusions) cultivated in continuous mode on glucose as the carbon source, imaged using STEM, magnification ×30,000. Picture taken by E. Ingolić, FELMI-ZFE Graz, and provided by M. Koller, University of Graz, Austria [83].

PHA, comprised of hydroxyalkanoic acid monomers, owes its uniqueness primarily to its fascinating production process. This process is a captivating biological phenomenon orchestrated by a diverse array of microorganisms such as *Alcaligenes latus*, *Azobacter vinelandii*, *Bacillus megaterium*, *Cupriavidus necator*, *Pseudomonas oleovorans*, and *Escherichia coli* [80]. These microorganisms naturally accumulate PHAs, especially polyhydroxybutyrate (PHB), as their principal lipid reserve within cellular membranes in the form of granules under conditions of metabolic stress (Figure 4) [81].

Biopolymer biosynthesis occurs at the cytoplasmic level when there is an excess of carbon and a deficiency of other vital nutrients required for cell proliferation, including phosphorus, nitrogen, and magnesium. Under such circumstances, intracellular accumulation of the biopolymer in granular form serves as a reservoir of carbon and energy [82]. 

This phenomenon mirrors the industrial emulsion polymerization process with remarkable precision. It serves as a captivating and exemplary instance of how certain biological processes can be seamlessly integrated into industries, catalyzing a new revolution centered around the effective harnessing of biological mechanisms. These mechanisms are renowned for their energy efficiency, high selectivity, and environmental friendliness, making them ideal candidates for integration into traditional industrial processes [83,84].

**Figure 5:**
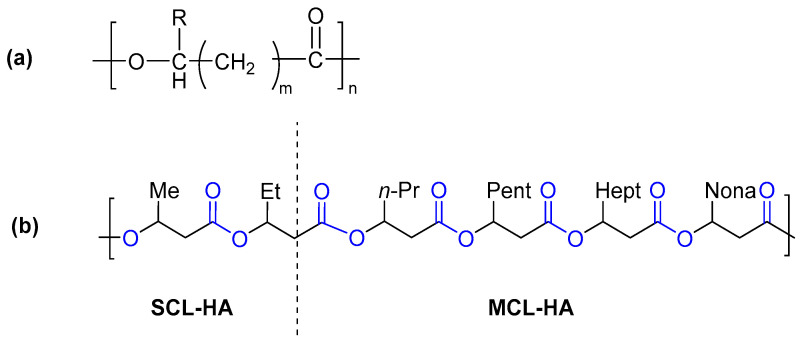
(**a**) General structure of polyhydroxy alkanoates (PHAs). Each monomer bears a side chain (-R). Usually, *m* can range from 1 to 8 carbon atoms, while *n* ranges from 100 to 3000 units. (**b**) Representation of some small-chain-length (SCL-HA) and medium-chain-length (MCL-HA) side groups. Adapted from reference [85].

From a structural point of view, polyhydroxy alcanoates are composed of hydroxylated fatty acid monomers that polymerize via ester bond formation with each monomeric unit bearing the alkylic chain as a side chain (Figure 5).

#### 2.2.1. Classification of Polyhydroxyoctanoate

PHA polymers are categorized into three groups based on their structure and molecular size: short chain length (SCL-PHA), medium chain length (MCL-PHA) and long chain length (LCL-HA) [84,85,86]. In terms of chain length, SCL-PHAs are the most commonly synthetized and studied [87].

Short-chain compounds consist of repeating 3 to 5 carbon building blocks (C_3_-C_5_). Some examples are poly (3-hydroxyvalerate) (PHV) and poly (3-hydroxybutyrate) (PHB), which is one of the most investigated PHA polymers. Medium-chain polymers (MCL-PHA, C_6_-C_14_) contain between 6 and 14 carbon monomers. A good example of such PHA is poly(3-hydroxyoctanoate) (PHO), which can be obtained from *Pseudomonas mendocina*. Lastly, long-chain PHAs encompass longer monomeric units (number of carbon atoms ≥ 15). They are the least common type of PHAs and are usually obtained from *Shewanella oneidensis* and *Aureispira marina* [84,85,86,88,89,90,91].

The differentiation in chain length/monomeric unit is achieved by selecting appropriate bacteria. Each microorganism has the ability to produce distinct types of monomeric units. This variance in chain size primarily stems from the substrate specificity of PHA synthases (PhaC): enzymes responsible for catalyzing hydroxy acids within a specific carbon length range and integrating them into the growing polymer chain [79,92].

For instance, *Cupriavidus necator* is renowned for synthesizing PHA plastics when provided with excess sugar substrate, with PHA accumulation levels reaching up to approximately 90% of the cell’s dry weight [93]. The PHA produced by this bacterium typically consists of monomers with a small number of carbon atoms, such as poly(3-hydroxyvalerate) (PHV) and poly(3-hydroxybutyrate) (PHB) [94]. On the other hand, *Pseudomonas mendocina* produces PHA composed of medium-molecular-weight monomers, imparting elastomeric properties and a relatively low mechanical strength [95]. In contrast, *Pseudomonas aeruginosa* synthesizes PHA composed of high-molecular-weight monomers, exemplified by poly(3-hydroxypentadecanoate).

#### 2.2.2. Biosynthesis of Polyhydroxy Alcanoates

The biosynthesis of PHAs is strictly linked to different kind of metabolic pathways. Among them, the most notorious is the one involved in the synthesis of PHB [96]. The method used to produce PHB involves starting from carbon sources such as glucose, sucrose, or lactose that are converted through different enzymatic reactions to acetyl-CoA. Under metabolic stress, acetyl-CoA enters the metabolic pathway of ketone bodies and is converted into PHB in three steps: 3-ketothiolase transform acetyl-CoA into acetoacetyl-CoA, which is then converted into (R)-3-hydroxybutyryl-CoA via the action of acetoacetyl-CoA reductase via a NADH-dependent mechanism of action. Finally, PHB synthase catalyze the polymer formation [81,97,98].

#### 2.2.3. Chemical and Physical Properties of Polyhydroxy Alcanoates

The monomeric composition of microbial PHAs is influenced by many factors, such as the strain of bacteria used, the composition of the growth medium, the carbon source, and the overall cultivation conditions. Consequently, PHA polymers can display a wide range of chemical, thermal, and mechanical properties, varying from rigid and brittle with a high crystallinity percentage to elastomeric polymers, thereby enabling diverse industrial applications. It is worth remembering that the properties of the polymeric material are closely related to the structure of the monomer (e.g., length of the side chain, length of the monomeric backbone, etc.). Therefore, the properties of PHA can be modified physically, chemically, or biologically to tune them to different applications [86,99,100].

Despite their potential for commercial use, many PHA-based materials still present some challenges that researchers need to tackle. For instance, poly(3-hydroxybutyrate) (PHB), one of the most-studied and characterized PHA-based polymers, is highly hydrophobic and exhibits low heat distortion temperatures and poor gas barrier properties. PHB, with its high crystallinity percentage (up to 80%), suffers from poor mechanical properties, making it unsuitable for various applications including packaging, biomedical and pharmaceutical uses like heart valves, vascular applications, and controlled drug delivery systems [101]. Additionally, PHA production costs are higher compared to other bio-based plastics, limiting their applications due to their undesirable physical properties.

**Figure 6:**
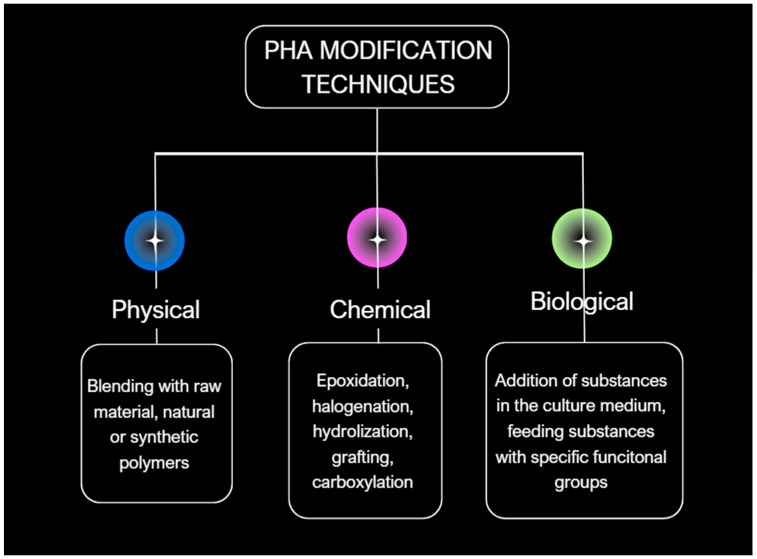
Graphical representation of the possible ways to achieve PHA modification [84,104].

Various approaches have been devised to enhance the physical and chemical properties of PHAs, aiming to overcome these limitations by modifying the biopolymers physically, chemically, or biologically to broaden their applications across different fields (Figure 6) [102,103]. There are several methods to tune and improve PHA properties, the most important of which are physical modification via blending with other polymeric materials and chemical modification of the polymeric chain. 

##### Blending

With regard to physical modification, blending PHA with natural raw materials and with synthetic biodegradable polymers is a highly studied technique in the literature. A polymer blend has been defined by IUPAC as “a macroscopically homogeneous mixture of two or more different polymers” [105]. In polymer technology, blending is a captivating solution that, by mixing a defined polymeric matrix with other substances, can allow researchers to obtain new polymeric materials with improved properties.

One of the major advantages of this approach to polymer modification relies in the possibility of tuning the properties of the original polymer to make it compatible with a specific and desired application [106]. Researchers have explored numerous low-cost, high-value raw materials and bio-based polymers as additives to modify PHA properties and create materials with enhanced characteristics. Some of the most notable examples include blending with natural polymers such as cellulose, starch, and lignin.

- Cellulose derivatives have garnered significant attention as viable blending agents with PHA due to their compatibility and capacity to accelerate PHA degradation rates. The incorporation of cellulose derivatives like cellulose acetate butyrate (CAB) has demonstrated notable enhancements in the various physical and mechanical properties of PHA, including but not limited to a PHB concentration-dependent increase in the T_g_ and an increase in the elongation at break of up to 7%. The utilization of PHA blends with cellulose derivatives has found diverse applications in the pharmaceutical and medical domains. These blends serve as effective carriers for poorly soluble drugs, facilitate blood coagulation processes, and are employed in pharmaceutical tablet coating, among other uses [107,108,109].
- Starch, being among the most promising natural polymers, boasts inherent biodegradability and widespread availability. Poly-3-hydroxybutyrate (PHB) stands out as the predominant type of PHA utilized thus far in formulating blends with starch [110]. The blending of PHA with starch or its derivatives has shown promise in reducing production costs while enhancing mechanical properties. Notably, studies have demonstrated the viability of blending thermoplastic starch with PHB. Incorporating thermoplastic starch at levels of up to 30 wt% with PHB has yielded improvements in tensile strength, Young’s modulus, and elongation at break when compared to pure PHB, all while yielding a more cost-effective material. These blends have expanded the potential applications of PHB as a coating material for food packaging, particularly on paper or cardboard substrates. Despite these benefits, blending PHAs with starch still presents challenges to tackle due to their inherent incompatibility, leading, for example, to difficulties in the production of non-brittle films [110,111,112].
- Lignin is a natural amorphous biopolymer made of repeating units of phenylpropane, featuring both aliphatic and aromatic hydroxyl groups, along with carboxylic acid groups [113]. The presence of these functional groups makes lignin a valuable material for blending purposes with PHA. It was demonstrated that lignin has a good miscibility with PHB, and lignin’s amorphous structure can decrease the formation of large spherulite crystals and secondary nucleation, which significantly affects the brittleness of PHB [114]. The studies of Kai et al. have shown that lignin and its butyrate derivative exhibit a high miscibility with PHB, and that it can affect PHB crystallization. In particular, it was demonstrated that lignin can effectively slow down PHB crystallization. These findings suggest that lignin fine powder could serve as a novel type of nucleating agent to modulate PHB crystallization [115]. The thermo-physical properties and rheology of PHB/lignin blends were deeply studied by Mousavioun et al. Their findings, based on TGA, DSC, and SEM analyses of the PHB/soda lignin blends, suggest that intermolecular interactions between PHB and soda lignin are favored at a soda lignin content of up to 40 wt%. These interactions are attributed to the formation of hydrogen bonds between the reactive functional groups of lignin and the carbonyl groups of PHB. While soda lignin enhances the overall thermal stability of PHB, it also reduces the initial decomposition temperature of PHB [116].

##### Chemical Modification

Chemical modification represents another promising solution for enhancing PHAs’ properties by introducing additional chemical groups into the PHA structure. Polyhydroxyalkanoates (PHAs) can undergo modifications through the incorporation of various chemical groups, leading to the development of chemically modified PHAs with enhanced functionalities. These modified PHAs hold potential for utilization as multifunctional materials. The most common chemical modifications involve carboxylation, halogenation, hydroxylation, epoxidation, and grafting processes on the PHA polymeric matrix.

The carboxylation process consists of the creation of -COOH functional groups, often mediated by KMnO_4_ as an oxidation agent [117]. This type of modification was demonstrated to be efficient in enhancing the hydrophilicity of the polymer [118]. The halogenation of PHA stands out as a remarkable method for enhancing the properties, functionalities, and applications of polymers.

The introduction of halogen atoms (such as chlorine, bromine, and fluorine) can be performed on both unsaturated and saturated PHA through addition or substitution reactions depending on the chemical characteristics of the substrate. This method offers a versatile means of broadening the functionalities and applications of the polymer. Depending on the quantity of halogen introduced, the resulting PHA demonstrates elevated melting and glass transition temperatures. However, this method also presents the drawback of reduced biocompatibility [119].

Hydroxylation presents a method to achieve a reduction in the molecular weight of the original polymer chain via hydrolysis reactions. This can proceed either through an acid- or base-catalyzed reaction and is usually carried out in the presence of low-molecular-weight mono or diol compounds. The most common methods employ either sodium hydroxide or para-toluene sulfonic acid (PTSA). PHA modified by these reactions might exhibit lower glass transition and melting temperatures [120].

Epoxidation represents a chemical modification method with the potential to enhance the thermal stability of PHA. This process involves converting double carbon–carbon bonds into epoxy groups. The high reactivity and facile conversion of epoxy groups into anionic and polar groups, even under mild conditions, underscore the significance of epoxidation as a pivotal strategy for PHA modification [121].

Grafting emerges as a highly reliable chemical modification method for PHA, wherein monomers are covalently bonded or “grafted” onto the polymer chain to achieve desired properties. This approach holds promise in preserving the inherent characteristics of PHA while introducing new ones. Various grafting methods are available, including radiation-based, enzyme-based, ionic, or radical grafting. Generally, this type of modification ensures minimal loss of original properties while imparting additional properties to the polymer [122,123].

#### 2.2.4. Applications of Polyhydroxyalcanoates

In terms of applications, PHAs can be employed across various fields, such as medicine, agriculture, and packaging, owing to their biodegradability, biocompatibility, and non-toxic nature.

PHA materials are extensively utilized in the medical industry owing to their excellent biodegradability and biocompatibility. PHA particles exhibit non-toxicity and are devoid of any pyrogenic, allergenic, carcinogenic, inflammatory, or teratogenic properties [124]. The employment of PHA beads has proven to be useful for medical and industrial applications such as protein purification [125], diagnostics and imaging [126,127], tissue engineering, etc.

In the medical field, PHA serves as a valuable material for drug delivery systems including nanoparticles and encapsulation of active pharmaceutical ingredients [128,129]. The in vivo and in vitro cytotoxicity of PHA carriers have been studied. A recent paper by Pevic et al. proved how the use MCL-PHA as a drug delivery system can both improve therapeutic effects and reduce systemic drug toxicity [130]. Bokrova et al. developed novel combined PHB–liposome nanoparticles and assessed their cytotoxicity in mammalian cells in vitro. The newly combined PHB–liposome particles demonstrated no toxicity to HEK (human embryonic kidney) and HaCaT (human immortalized keratinocyte) cells [131]. These PHB nanoparticles (NPs) exhibit potential as active carriers for both hydrophobic and hydrophilic active ingredients, including antioxidants, anti-aging compounds, complex natural extracts, and antibacterial agents [124]. PHA particles offer versatility in modifications through various surface-binding proteins, thereby expanding their practical applications and reducing PHA nanoparticle cytotoxicity [132]. In a study by Fan et al., the natural PHA binding protein PhaP was effectively adsorbed onto PHB NPs through hydrophobic interactions and used as a drug delivery system for targeted accumulation in prostate tumors. This system allowed for a remarkably enhanced cellular uptake in the human prostate cancer cell line PC3 compared to non-functionalized NPs. This study demonstrates the potential of surface-modified PHA nanoparticles to enhance or target phagocytosis, thus paving the way for their potential clinical application in the future [133].

Spherical polyhydroxyalkanoates have also found many applications in the vaccine engineering field [134]. Given that PHB has received approval from the U.S. Food and Drug Administration (FDA) for clinical studies, and that its 3-hydroxybutyric acid component is a natural constituent of human blood, this type of PHA has been proposed as a safe option for use as an antigen carrier in vaccine formulations [135,136].

PHAs have been studied and used to produce PHA bead-based particles embodied with specific antigens as vaccines formulations against different biological agents such as *Pseudomonas aeruginosa* [137], *Mycobacterium tuberculosis* [138,139,140,141], *Streptococcus pneumoniae* [142,143,144], and *Neisseria meningitidis* [145]. Additionally, PHA is utilized in tissue engineering to fabricate scaffolds for regenerative medicine purposes [146,147,148]. PHA is also employed in the agricultural field, not only as an encapsulating agent for pesticides and to control their release kinetics but also as a seed-protecting agent [149]. The packaging and food service industries utilize PHA due to its biodegradability and permeability properties, making it an attractive alternative to petroleum-based plastics. PHA-based packaging films are utilized in the production of plastic bags, cups, and similar products [150].

### 2.3. Polyhydroxy Polyamides

**Scheme 2:**
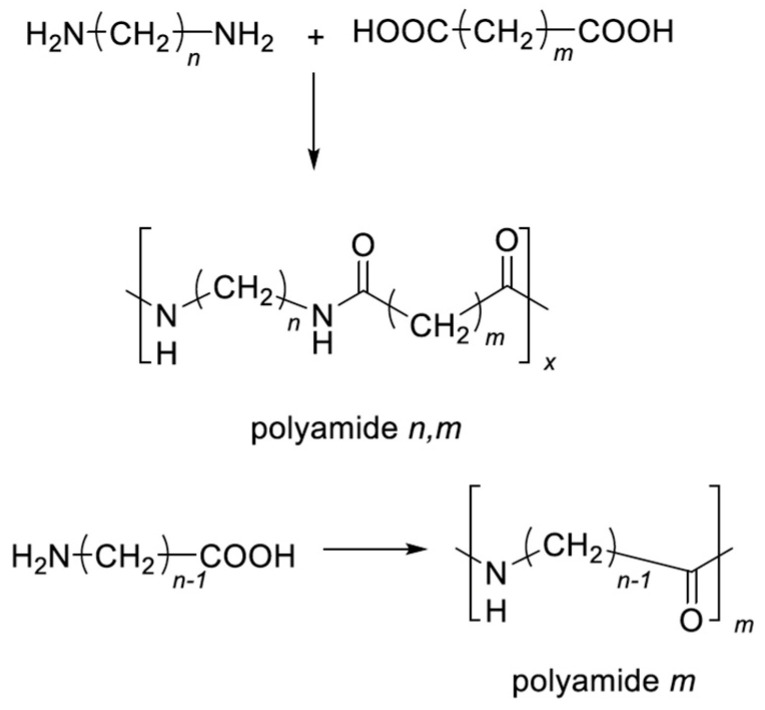
Polyamide types.

A polyamide, by definition, is a condensation product in which the monomers are linked via an amide bond. Perhaps the most renowned polyamide is Nylon-6,6, which has led to polyamides being commonly referred to as “nylons” in a general sense. Linear polyamides are formed through a condensation reaction between bifunctional monomers. When amino acids or their lactam forms are used as monomers, the resulting polymer is classified as an AB type polymer, with A representing the amino group and B the carboxylic group. The resulting polyamide is denoted as nylon-*n*, with *n* representing the number of carbon atoms between the amino and carboxylic termini. Similarly, polymers resulting from the reaction between a diamine and a dicarboxylic acid are classified as AABB type, with two numbers (*m*, *n*) indicating the number of carbon atoms separating the amino and carboxylic functional groups, respectively (Scheme 2) [151].

The physical properties of nylons are closely tied to the distance between the functional groups and thus the length of the polyethylene chain. Consequently, common nylons exhibit very similar performances, displaying a “monotonous behavior”, in contrast to natural polymers, which exhibit greater diversity in their physical, chemical, and biological functions [152]. This has led to the gradual replacement of these polymers with high-performance materials ad hoc designed to express improved biocompatibility, biodegradability, and other desired properties [153].

Efforts have already been made to synthesize biodegradable nylons, with findings indicating that polyamides containing methyl and hydroxyl groups demonstrate biodegradability. Furthermore, the presence of electron-withdrawing substituents near the carbonyl groups has been shown to enhance their hydrolysis rate [154]. Biodegradable synthetic polymers are typically designed to mimic natural peptides or proteins to enhance both chemical and enzymatic hydrolysis of the final product. From a synthetic perspective, introducing asymmetric carbons into the polyamide backbone allows for control over the physical properties of the polymer by modulating the tacticity of the macromolecule [155].

Carbohydrate-based synthetic polymers can be prepared through the polymerization reaction of appropriate activated monomers. Overall, the interest in polyaldaramides has increased in the past decade. Polyaldaramides, also known as polyhydroxypolyamides (PHPAs) or hydroxylated nylons, are hydroxylated, linear polyamides of the AABB type, where the diacid monomer units of typical nylons are replaced by an aldaric acid. Aldaric acids are dicarboxylic acids derived from the oxidation of aldoses. Their chemical nature and renewable origin make them promising candidates as monomers or building blocks for the synthesis of biobased polymers, particularly hydroxylated polyesters and polyamides [156,157]. In terms of environmental impact, these compounds have garnered significant attention due to the renewable nature of the carbohydrate backbone of the monomer unit and their high degradability in soil, especially when compared with traditional nylons or poly(ethylene terephthalate) [158,159].

**Scheme 3:**
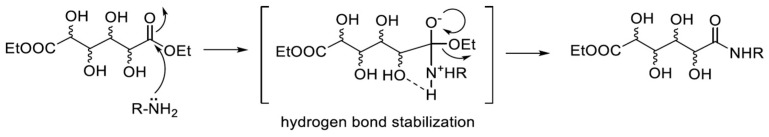
Hydrogen bond stabilization of the intermediate reaction stage.

The regio- and stereochemical properties of the macromolecule depend on the configuration of the chosen monomers. Although derivatives of aldaric acids could have been employed in polymerization reactions, their application in this regard was not investigated until the 1950s. In fact, the first example of these polymers was reported by Wolform et al. in 1958 [159], and later on, more in-depth studies of these polymers were carried out by Ogata et al. in Japan in the 1970s. The pioneering work by Ogata et al. demonstrated that the condensation between a diethyl ester of an aldaric acid (such as tartaric or galactaric) and a diamine could proceed under mild conditions. It was found that the presence of heteroatomic groups (such as ether or hydroxyl functional groups) in the α- or β-position to the ester group greatly enhanced the reactivity of the diester monomer in polymerization reactions carried out in polar solvents [158,160,161]. The polymerization of diethyl galactarate with hexamethylenediamine yielded a hydroxylated analogue of nylon-6,6 that did not melt or decompose at 200 °C. These reactions were performed in solvents such as methanol, dimethyl sulfoxide, and N-methylpyrrolidone under mild conditions. The increased reactivity of esters toward aminolysis due to the presence of the hydroxyl group was attributed to hydrogen bonding of the -OH group with the approaching amino group in the intermediate reaction stage (Scheme 3).

**Scheme 4:**
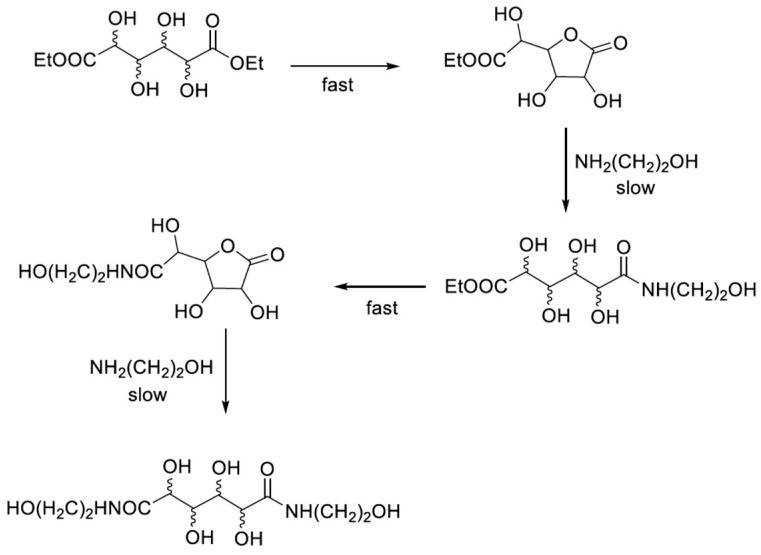
General aminolysis pathway demonstrated by Hoagland et al. [162].

Further examination of this reaction was carried out by Hoagland et al., who investigated the reaction mechanisms of aminolysis of six-carbon galactaric acid diesters [162]. They found that the aminolysis proceeds through a two-step sequence: a fast, base-catalyzed lactonization to give the γ-lactone intermediate, followed by the slow aminolysis of the lactone (Scheme 4).

The lactonization of the ester appears to be much faster than the aminolysis of the ester. This study by Hoagland et al. demonstrates that the activation of the aldaric acid diesters is due to the easy formation of the highly reactive five-membered aldarolactones [162].

Polyaldaramides are typically prepared using aldaric acid monomers that are activated as esters or acyl chlorides, often with acetylated protected hydroxyl groups. Polyamides with free hydroxyl groups are then obtained via deacylation in aqueous ammonia. The synthesis of unprotected activated d-glucaric acid dimethyl or diethyl ester monomers and their polymerization were first investigated and patented by Kiely et al. [163,164]. The initial polyhydroxypolyamide (PHPA) reported by Kiely was prepared under Fisher esterification conditions in methanol. Subsequent reports on the synthesis of d-glucaric acid-based PHPAs have focused on the use of methyl d-glucaro-1,4 lactone as the starting monomer [165]. Carbohydrate-derived polyamides exhibit interesting properties, particularly in terms of crystallinity, which is typically associated with chain stereoregularity. AABB-type polyamides obtained through conventional polycondensation are stereoregular when their monomers have a 2-fold axis of symmetry. Otherwise, they may enter the polymer chain in two opposite orientations, giving rise to non-stereoregular polyamides. The polyglucaramides obtained using the above-mentioned protocol are termed *stereo-random* because d-glucaric acid does not have a symmetrical structure and therefore gives rise to randomly oriented glucaric acid units in the backbone of the macromolecule [166]. In fact, such symmetry restriction is met only by the aldaric- or alditol-based monomers having *threo-*, *manno-*, and *ido*-configuration. Nonetheless, stereoregular AABB-type polyamides derived from non-centrosymmetric d-glucaric acid have been prepared by Kiely et al. using synthetic methods that can differentiate between the reactivity toward the aliphatic diamine of the aldaric acid’s two carboxyl groups [157].

**Scheme 5:**
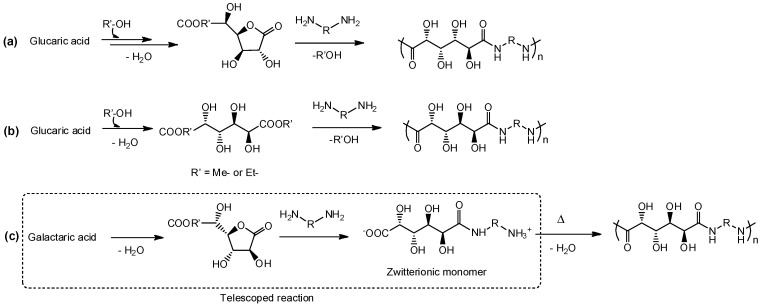
Synthetic procedure for the synthesis of polyhydroxy polyamides, modified from reference [167], (**a**) synthetic path involving protection and lactone formation first, (**b**) polycondensation using diester monomers and (**c**) more recent polymerization technique using zwitterionic monomers.

While efforts have been focused on understanding the chemical behavior and finding convenient synthesis pathways for PHPAs derived from d-glucaric acid, meso-xylaric acid, and l-mannaric acid, little investment has been made in using galactaric acid derivatives as starting monomers (Scheme 5).

Only a few examples of poly(galactaramides) being synthesized and partially characterized are present in the literature. Usually, the most convenient method reported for the synthesis of polyhydroxy polyamides from galactaric acid is using galactaric acid esters as starting monomers (Scheme 5b) [168,169,170]. With the aim of avoiding the use of protective groups and favoring a rigorous 1:1 diacid-diamine ratio control, Gambarotti et al. reported the synthesis of a new zwitterionic monoamide of galactaric acid to be used as a building block in the polymerization reaction (Scheme 5c). This protocol involved the conversion of galactaric acid into the corresponding γ-galactaro lactone first, followed by its reaction with diamines leading to the formation of the corresponding zwitterionic monoamides with good yields and selectivity. This new class of monomers was then employed in polymerization to obtain the corresponding poly(galactaramides), showing a high atom economy and comparable degree of polymerization (DP) usually obtained by using the protected galactaric ester form [167].

In terms of application, polyaldaramides, which appear to be biocompatible and biodegradable, offer easy access to a wide range of polymers that can be applied as biodegradable adhesives, timed release fertilizers, industrial chemicals for the textile and paper industries, water treatment chemicals, detergent components, hydrogel components, and film and fiber materials [171]. Also, an interesting study was published demonstrating that poly(glucaramides) can form a nanoparticulate system that can be used for slow and controlled release applications [172].

## 3. Conclusions

In conclusion, the exploration of bio-based polymers such as PLA, PHA, and polyhydroxy polyamides unveils a promising frontier in sustainable material science. These polymers offer a compelling alternative to traditional petroleum-based plastics, exhibiting comparable or even superior properties while significantly reducing environmental impacts. Their versatility and compatibility with various applications ranging from packaging to biomedical devices underscore their potential to revolutionize the polymeric material sector and industry. While challenges such as cost-effectiveness, scalability, and processability remain, ongoing research and technological advancements continue to drive innovation in this field. As we move towards a more sustainable future, the adoption of bio-based polymers stands as a crucial step in mitigating plastic pollution and fostering a greener, more resilient global economy. Through collaborative efforts between academia, industry, and policymakers, the integration of these eco-friendly materials into mainstream usage holds the promise of a brighter, more sustainable tomorrow.

## Figures and Tables

**Table 1:** Comparison between the physical properties of PLA [28,29,30], polypropylene (PP) [31,32,33,34], and high-density polyethylene (HDPE) [34,35,36,37,38].

Properties	Type of Polymer				
	PLA	PLLA	PDLLA	PP Homopolymer	HDPE
Polymer density—ρ (g/cm^3^)	1.21–1.25	1.24–1.30	1.25–1.27	0.91–0.94	0.80–1.80
Tensil strength—σ (MPa)	21–60	15.5–150	27.6–50	22–34.4	11.6–40
Young’s modulus—E (GPa)	0.35–3.5	2.7–4.14	1–3.45	0.545	0.30–1.30
Strain—ε (%)	2.5–6	3.0–10.0	2.0–10.0	//	20
Elongation at break (%)	10	//	//	3–700	3.5–800
Glass transition temperature—T_g_ (°C)	45–60	55–65	50–60	−25	−120
Melting temperature—T_m_ (°C)	150–162	170–220	n.a. ^1^	160–166	127–255
Crystallinity (%)	42	//	//	30–60	70

^1^ n.a.—not applicable: amorphous material does not have a well-defined melting point.

## Data Availability

Not applicable.
